# Changing perceptions of weight in Great Britain: comparison of two population surveys

**DOI:** 10.1136/bmj.a494

**Published:** 2008-07-10

**Authors:** F Johnson, L Cooke, H Croker, Jane Wardle

**Affiliations:** 1 Cancer Research UK Health Behaviour Research Centre, Department of Epidemiology and Public Health, University College London, London WC1E 6BT

## Abstract

**Objectives** To examine changes in public perceptions of overweight in Great Britain over an eight year period.

**Design** Comparison of data on self perceived weight from population surveys in 1999 and 2007.

**Setting** Household surveys of two representative samples in Great Britain.

**Participants** 853 men and 944 women in 1999, and 847 men and 989 women in 2007.

**Main outcome measures** Participants were asked to report their weight and height and classify their body size on a scale from “very underweight” to “obese.”

**Results** Self reported weights increased dramatically over time, but the weight at which people perceived themselves to be overweight also rose significantly. In 1999, 81% of overweight participants correctly identified themselves as overweight compared with 75% in 2007, demonstrating a decrease in sensitivity in the self diagnosis of overweight.

**Conclusions** Despite media and health campaigns aiming to raise awareness of healthy weight, increasing numbers of overweight people fail to recognise that their weight is a cause for concern. This makes it less likely that they will see calls for weight control as personally relevant.

## Introduction

Inaccurate recognition of weight status is a threat to healthy weight control. Until the mid-1990s, the emphasis was on young women’s tendency to identify themselves as overweight despite a healthy body size,^1^ ^2^ ^3^ and concern focused primarily on the risks of eating disorders. With rates of anorexia remaining stable^4^ but obesity rates rising inexorably,^5^ ^6^ attention has now turned to awareness of weight status among those who are overweight or obese. A considerable proportion of overweight adults—men in particular—do not recognise that their body weight is too high,^7^ ^8^ ^9^ and many parents fail to recognise that their children are overweight.^10^ ^11^ ^12^

The clinical categories “overweight” and “obese,” defined by BMI (body mass index) thresholds of over 25 and over 30, respectively, are used universally by health professionals to evaluate risks associated with excess body weight. Lay definitions of these terms, however, might differ from those of clinicians, and such discrepancies can present a barrier to communication between the health profession and the public. The public’s weight perceptions are probably less rigidly defined and influenced by perceptions of acceptable weight related to specific cultural and social groups.^13^ ^14^ ^15^

Changes in the social environment over recent years could have affected weight perceptions in several ways. Increased attention to the “obesity epidemic” and publicity channelled through the media and health professionals to encourage appropriate action for weight control^16^ ^17^ might be expected to promote recognition of overweight. There has also been an emphasis on positive body images for young women, which should have reduced inaccurate perceptions of overweight among normal weight women. On this basis, weight recognition should have become more accurate.

Media reports about body weight, however, often use images of severe obesity, which could give the impression that extremely high weights are required to meet medical criteria for overweight. In addition, increases in adiposity in the population might have “normalised” overweight, leading to increased acceptance of body fat and reduced recognition of excess weight. The social comparison effects might also mean that fewer normal weight individuals incorrectly perceive themselves to be overweight. On this basis, recognition of overweight might be expected to be worse in overweight and obese individuals.

Accuracy in self diagnosing overweight can be approached with the diagnostic concepts of sensitivity and specificity.^18^ Sensitivity is the proportion of truly overweight people who identify themselves as such, while specificity is the proportion of people who are not overweight who identify themselves correctly as not overweight. If the combined emphasis on public awareness of the risks of obesity and promotion of a healthy body image in young women has been successful, then both sensitivity and specificity of self diagnosed overweight should have increased. On the other hand, if social comparison processes have led to normalisation of overweight, any increase in specificity might have been accompanied by a decrease in sensitivity.

We investigated changes in public perception of overweight over an eight year period, and assessed effects on the self diagnostic abilities of overweight and normal weight British adults.

## Methods

### Study design and participants

We compared self reported weights and perceptions of weight from two population based surveys carried out eight years apart. The first survey was carried out through the Office for National Statistics (ONS) omnibus survey of March 1999. A probability sample of women and men was selected, using random sampling of addresses on the postcode address file of private households in Great Britain. Further details of the methods can be obtained at www.ons.gov.uk/about/who-we-are/our-services/omnibus-survey. Within each household, one adult was randomly selected for interview. These data have been previously published.^8^

The second survey was a face to face omnibus survey conducted in May 2007 by the British Market Research Bureau (BMRB) using a two stage random location sampling method. Enumeration districts defined by the 2001 census (excluding Northern Ireland and the Western Isles) were selected at random, and 83 sample areas were used. Sample units, composed of around 300 households, were stratified by demographic characteristics and region and randomly selected, with probability of selection proportional to the population. The use of stratifiers ensures all types of area are fully represented. Further information is available from www.bmrb.co.uk/?id=755. In both surveys the interviews were undertaken in the home with only one interview per household. Both produced samples that closely resembled the demography of the population of Great Britain.

### Measures

*Demographic variables*—Demographic variables included in the present analyses were age, sex, and age on leaving education.

*Anthropometric data*—Weight and height were self reported in whichever metric the individual preferred. Use of self reported anthropometric data means that height is likely to be overestimated and weight underestimated,^19^ ^20^ and therefore average BMI and the proportion of the population who are overweight or obese will be underestimated in both samples. Participants were divided into weight groups using BMI cut offs of <18.5 (underweight), >25 (overweight), and >30 (obese).

*Perceived weight*—Participants were asked to select a descriptor for their own body weight from the following list: very underweight, underweight, about right, overweight, and very overweight. The 2007 survey also included the category “obese.” For most of the analyses reported here, we dichotomised the data into a “perceived overweight” group, comprising the top two (three in 2007) categories, versus the rest.

### Data analysis

Data were provided with weightings for household size and analyses were carried out on weighted and unweighted data, but as there were no substantial differences in results, we present the unweighted results. Analyses were carried out in SPSS version 14 and Stata version 9.2. We used *t* tests and χ^2^ analyses for comparisons between the two surveys and further examined perceptions of overweight using log binomial regression, with dichotomised perceived overweight as the dependent variable. Independent variables were age, age on leaving education, sex, survey year, and weight group. We calculated specificity and sensitivity of perceptions of overweight and 95% confidence intervals using the efficient score method (corrected for continuity), as described by Newcombe.^21^

## Results

### Sample characteristics, 1999 and 2007

In 1999, 1894 interviews were carried out, comprising 882 men and 1012 women. Adequate weight and height data were collected from 853 men and 944 women. The 2007 sample of 1998 participants comprised 895 men and 1103 women, of whom 847 men and 989 women provided adequate data on height and weight.

There was no significant difference in sex balance between the two time points: 53% women in 1999 and 54% women in 2007. There was no overall difference in age, but women in the 1999 sample were slightly older than those in the 2007 sample (*t*=3.05, P<0.01) (table 1). There was a significant difference in age of completing education (*t*=2.61, P<0.01) with participants in the 2007 sample being slightly older. This was probably because of increases in the legal school leaving age.

**Table 1 tbl1:** Participants in survey samples, 1999 and 2007. Figures are means (SD)

	All participants			Women			Men	
	1999 (n=1797)	2007 (n=1836)		1999 (n=944)	2007 (n=989)		1999 (n=853)	2007 (n=847)
Age	48.25 (18.36)	47.61 (19.04)		49.32 (18.75)	46.74 (18.53)		47.06 (17.86)	48.64 (19.57)
Age on leaving education	17.07 (2.83)	17.32 (2.96)		17.03 (2.76)	17.28 (2.89)		17.11 (2.90)	17.38 (3.03)
Height (cm)	169.15 (10.18)	168.89 (10.38)		162.69 (7.32)	162.74 (7.72)		176.29 (7.87)	176.07 (8.27)
Weight (kg)	71.91 (14.89)	74.91 (16.19)		65.19 (13.20)	68.31 (14.44)		78.55 (13.42)	81.55 (15.12)
BMI	24.91 (4.48)	26.17 (5.22)		24.53 (4.81)	26.05 (5.63)		25.33 (4.03)	26.30 (4.71)

### Weight and perceptions of overweight

Height did not differ significantly between the samples, but both weight and BMI were higher in 2007 (*t*=6.09, P<0.001, and *t*=7.77, P<0.001, respectively). The proportion of respondents whose BMI placed them in the obese category had nearly doubled, from 11% to 19% (table 2).

**Table 2 tbl2:** Self reported and perceived weight 1999 and 2007. Figures are percentages (numbers)

	All participants			Women			Men	
	1999	2007		1999	2007		1999	2007
Reported weight status*:								
Underweight	2.8 (50)	2.9 (53)		3.9 (37)	3.3 (33)		1.5 (13)	2.4 (20)
Normal weight	54.5 (979)	44.2 (812)		59.6 (563)	47.2 (467)		48.8 (416)	40.7 (345)
Overweight	31.9 (574)	34.2 (628)		24.8 (234)	30.9 (306)		39.9 (340)	38.0 (322)
Obese	10.8 (194)	18.7 (343)		11.7 (110)	18.5 (183)		9.8 (84)	18.9 (160)
Perceived weight:								
Underweight†	7.6 (136)	5.1 (94)		6.9 (65)	3.3 (33)		8.3 (71)	7.2 (61)
About right weight	45.7 (821)	47.3 (869)		43.9 (414)	44.5 (440)		47.7 (407)	50.6 (429)
Somewhat overweight	39.3 (706)	38.5 (706)		39.6 (374)	40.3 (399)		38.9 (332)	36.2 (307)
Very overweight	7.5 (134)	7.3 (134)		9.6 (91)	9.1 (90)		5.0 (43)	5.2 (44)
Obese	NA	1.8 (33)		NA	2.7 (27)		NA	0.7 (6)

NA=not applicable (not a category in 1999 survey).

*Mutually exclusive categories.

†Includes very underweight.

In contrast with the upward trends in overweight and obesity, trends in perceived overweight were downward. In 1999, 43% of the population had a BMI that put them in the overweight or obese range, of whom 81% perceived themselves to be overweight or very overweight. In 2007, 53% of the population had a BMI in the overweight or obese range, of whom only 75% reported themselves to be overweight, very overweight, or obese.

We used log binomial regression to establish the significance of differences in weight perceptions between 1999 and 2007, controlling for differences in demographic composition of the samples (table 3). Age on leaving education did not achieve significance in the model. All other independent variables were significant predictors of perceived overweight.

**Table 3 tbl3:** Log binomial regression: variables associated with perceived overweight

	Relative risk of perceived overweight (95% CI)	z score	P value
Age	0.998 (0.996 to 0.999)	−2.54	0.011
Age on leaving education	0.997 (0.994 to 1.000)	−1.92	0.055
Weight group (BMI):			
Underweight (<18.5)	0.05 (0.01 to 0.34)	−3.03	0.002
Normal weight (18.5-<25)	1.00	—	—
Overweight (25-<30)	3.69 (3.35 to 4.07)	26.26	<0.001
Obese (>30)	4.82 (4.39 to 5.31)	32.29	<0.001
Sex:			
Men	1.00	—	—
Women	1.33 (1.26 to 1.40)	10.20	<0.001
Survey year:			
1999	1.00	—	—
2000	0.87 (0.83 to 0.92)	−4.86	<0.001

The effect of survey year on perception of overweight was highly significant, with participants in 2007 less likely to perceive themselves as overweight, given their weight group, sex, age, and education. Figure 1 shows how perceptions of overweight changed across the BMI spectrum and between the two surveys.

**Figure fig1:**
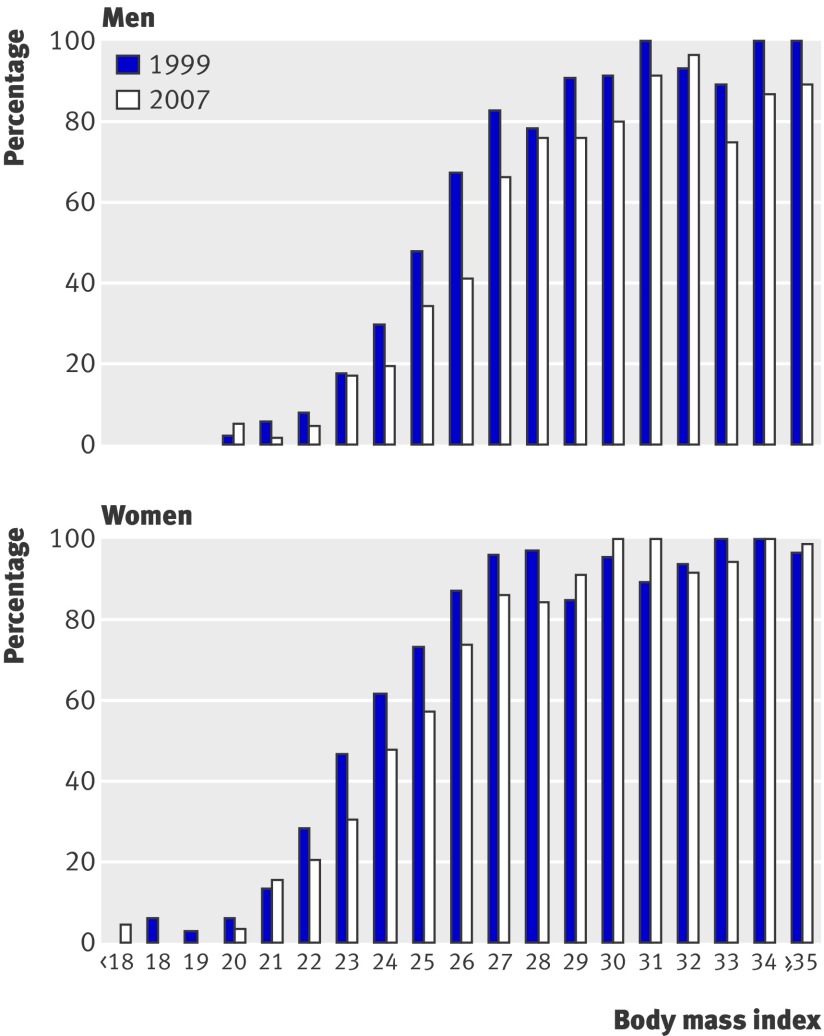
**Fig 1** Proportion of men and women who perceived themselves overweight. All BMI values rounded down

Weight group was strongly associated with perceived overweight. Just one (<1%) of the underweight participants and 19% of normal weight participants perceived themselves to be overweight, compared with 70% of those who were overweight and 94% of those who were obese.

### Sensitivity and specificity of self perception of overweight 

We examined sensitivity and specificity of weight perceptions, together with 95% confidence intervals, for the two samples (table 4). In the sample as a whole, sensitivity of recognition of overweight decreased between 1999 and 2007, alongside an increase in specificity. When we analysed data for men and women separately, we found a similar pattern of results for both groups, but results for the men did not reach significance. Figure 2 shows changes in sensitivity and specificity[Fig fig2].

**Table 4 tbl4:** Prevalence of overweight and sensitivity and specificity of recognition of overweight in men and women, 1999 and 2007

	Prevalence/sensitivity/specificity (95% CI), No in group		χ^2^, P value
	1999	2007	
**Prevalence**			
Men	0.50 (0.46 to 0.53), 903	0.57 (0.54 to 0.60), 916	7.72, P=0.005
Women	0.36 (0.33 to 0.40), 895	0.49 (0.46 to 0.53), 921	25.65, P<0.001
All	0.43 (0.40 to 0.45), 1798	0.53 (0.51 to 0.55), 1837	30.15, P<0.001
**Sensitivity**			
Men	0.75 (0.70 to 0.79), 446	0.67 (0.62 to 0.71), 512	3.09, P=0.079
Women	0.90 (0.86 to 0.93), 331	0.83 (0.80 to 0.87), 449	10.40, P=0.001
All	0.81 (0.78 to 0.84), 777	0.75 (0.72 to 0.78), 961	8.02, P=0.005
**Specificity**			
Men	0.86 (0.83 to 0.89), 457	0.90 (0.87 to 0.93), 404	3.51, P=0.061
Women	0.74 (0.70 to 0.77), 564	0.78 (0.75 to 0.82), 472	6.47, P=0.011
All	0.79 (0.76 to 0.81), 1012	0.83 (0.81 to 0.86), 876	9.87, P=0.002

**Figure fig2:**
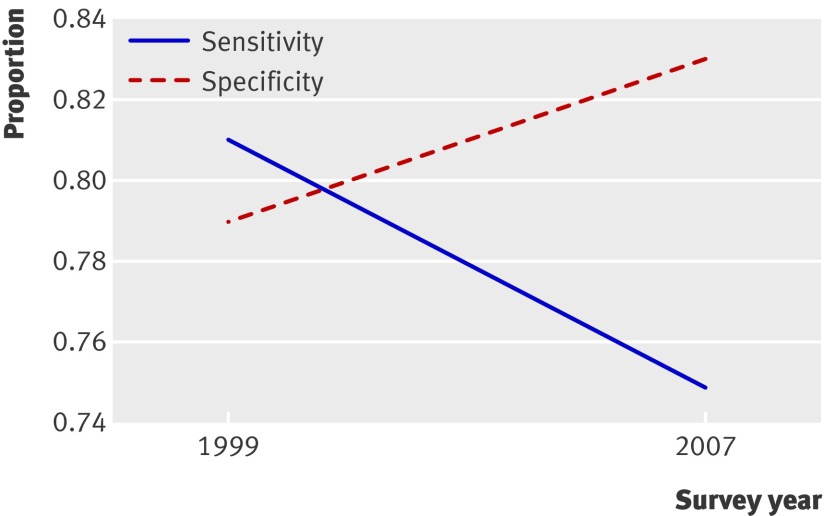
**Fig 2** Sensitivity and specificity of perception of overweight. Sensitivity denotes proportion of overweight participants who correctly identify themselves as overweight. Specificity denotes proportion of normal and underweight participants who correctly identify that they are not overweight

## Discussion

Despite the topic of weight scarcely being out of the news, these data from two population surveys show that fewer overweight and obese people defined themselves as overweight in 2007 than in 1999. The changes indicate a marked decline in sensitivity with respect to individuals’ detection of their own overweight. There was a concurrent improvement in specificity, with fewer people of normal or low weight believing themselves to be overweight. These effects were strongest in women, marginally failing to reach significance in men.

### Interpretation

A decline in sensitivity of recognition of overweight has important implications for the targeting of public health messages, which are unlikely to reach marginally overweight individuals if they fail to identify themselves as targets. These are the very people for whom lifestyle changes might have beneficial effects, potentially preventing weight related comorbidities. Recent research in primary care has suggested that communication between primary care practitioners and overweight patients is inadequate,^22^ and our results show on a national level that the attempts of health professionals to ensure that overweight individuals are aware of their weight status have been largely unsuccessful.

Increased attention to the health risks of excess weight might have left individuals more reluctant to identify themselves with labels such as “overweight” or “obese.” Certainly, there is evidence that some overweight individuals resist identifying with terminology that they perceive as stigmatising, preferring to adopt euphemistic identifiers for overweight such as “chubby” or “big boned.”^23^ This raises the question of how health professionals can best establish a vocabulary for the discussion of body weight that is precise enough and neither minimises the risks associated with excess weight nor provokes disengagement on the part of the patient.

One advantage of the shifting standard for overweight is that slightly fewer normal weight women think they are overweight. This has implications for practitioners and policy makers working in the field of eating disorders as well as obesity prevention. Concern has often been expressed that women are unnecessarily worried about their weight.^24^ ^25^ Our data suggest that inappropriate perceptions of overweight are declining among women in the normal weight range.

### Explaining changing weight perceptions

Various factors might have contributed to the declining ability of overweight individuals to recognise that their weight is too high. Social comparison is likely to play an important role in the development of societal weight norms, resulting in the threshold for perceived overweight rising in line with increasing weight in the population. International data have suggested that perceptions of overweight are related to levels of overweight in the local population, supporting the social norm hypothesis.^14^ In the context of changing population weight, a greater understanding of the role of social comparison in weight perception would be beneficial.

Another possible explanation relates to the type of images that often accompany media and health information. Photographic illustrations often depict severely obese people, untypical of the overweight population. This might act as false reassurance for those who are “merely” overweight, implicitly reinforcing a perception that messages about healthy eating and exercise are not aimed at them.

### Strengths and weaknesses of the study

While the demographic composition of the two samples was similar, there were small differences between years. Women in the 2007 survey were slightly older, and both men and women report more years of schooling. Inclusion of these as covariates in the analyses, however, did not change the findings. Data collection methods were not identical between the two surveys. In 2007, the option for university researchers to include items in the ONS Omnibus survey was not available, and therefore we used the BMRB Omnibus survey. Both samples produced were representative of the population and in both cases a computer assisted face to face survey was used, and therefore any social desirability bias is likely to affect both sets of data in a similar way.

A drawback of the methods is the use of self reported heights and weights. The use of self reports facilitates large scale data collection but is always a source of inaccuracy, resulting in underestimates of weight (particularly in women) and overestimates of height (particularly in men).^19^ ^20^ It is therefore likely that BMI and the prevalence of overweight are underestimated in both samples. There is no reason to expect that this accounts for the difference in weight perceptions as the same methods were used in both surveys and any inaccuracy is likely to be similar across both phases of data collection. In the absence of a study comparing self reported and measured weights and height over time, however, it is not possible to be certain that there has been no change in estimations of weight and height.

**What is already known on this topic**

Perceptions of overweight in the population do not correspond well to the definitions used by health professionals
Many overweight and obese individuals fail to recognise that their weight is too high

**What this study adds**

As the proportion of overweight people in Great Britain has increased, the ability of overweight individuals to “self diagnose” their weight problem has declined

## Notes

Data collection was carried out by the Office for National Statistics (ONS) and the British Market Research Bureau (BMRB).

Contributors: JW was the lead researcher and conceived the project. She led the study design and development of materials in both 1999 and 2007 and participated in data analysis and interpretation for both surveys. She made a substantial contribution to content of the paper and is guarantor. FJ participated in the study design and development of materials for the 1999 survey, carried out statistical analyses of data from both surveys, and drafted the paper. LC designed materials and liaised with the data collection bodies in 2007. She also made revisions to the paper. HC was involved in the development of survey materials in 2007 and made revisions to the paper. All authors approved the final version of the paper for publication.

Ethical approval: Not required.

Provenance and peer review: Not commissioned; externally peer reviewed.

## Notes

**Cite this as:** *BMJ* 2008;337:a494
